# Editorial: Advancing equity in allergic disease management through innovative clinical strategies

**DOI:** 10.3389/falgy.2026.1918802

**Published:** 2026-07-20

**Authors:** Ayobami Akenroye, Akilah A. Jefferson, Sharmilee M. Nyenhuis

**Affiliations:** 1 Division of Allergy and Clinical Immunology and Channing Division of Network Medicine, Department of Medicine, Brigham & Women’s Hospital, Boston, MA, United States; 2 Harvard Medical School, Boston, MA, United States; 3 Health Systems Science, Kaiser Permanente Bernard J. Tyson School of Medicine, Pasadena, CA, United States; 4 Section of Allergy, Immunology and Pediatric Pulmonology, Department of Pediatrics, University of Chicago, Chicago, IL, United States

**Keywords:** allergic diseases, asthma, atopy, equity, LMIC

Allergic diseases affect billions of people worldwide, yet the benefits of advances in diagnosis, therapeutics, and disease management have not been distributed equally. Across conditions ranging from allergic rhinitis and asthma to hereditary angioedema and primary immunodeficiencies, patients, especially those in low-to-middle income countries or underserved populations in developed countries, continue to experience disparities in access, diagnosis, treatment, and outcomes. These inequities are driven by a complex interplay of social determinants of health, healthcare infrastructure, workforce limitations, environmental exposures, and policy decisions. While scientific innovation has transformed many aspects of allergy and immunology, innovation alone does not improve outcomes. The challenge before clinicians, researchers, health systems, and policymakers is ensuring that effective interventions reach the patients who need them most.

The six articles in this Research Topic highlight this important reality. Together, they demonstrate that advancing equity in allergic disease management requires more than the development of new therapies and technologies. It requires patient education, implementation science, digital innovation, healthcare delivery redesign, and policy interventions that make evidence-based care accessible, affordable, and sustainable. Although the contributing articles address different diseases and settings, they converge around a common theme: meaningful improvements in allergic disease outcomes occur when innovation is paired with implementation and when care is made easily accessible to patients.

A fundamental component of equitable care is ensuring that patients understand their disease. In their cross-sectional survey of patients with allergic and non-allergic rhinitis in China, Su and colleagues examined knowledge, attitudes, and practices surrounding rhinitis. While patients with allergic rhinitis generally demonstrated greater disease-related knowledge than individuals with non- allergic rhinitis, important knowledge gaps persisted in both groups. These gaps were particularly notable regarding environmental exposures and the impact of humidity and indoor environmental factors. These findings are important because asthma and allergic diseases are often heavily influenced by environmental factors. Effective management requires more than prescribing medications; it requires helping patients understand the exposures and behaviors that contribute to symptoms and disease control. The study also highlights differences between allergic and non-allergic rhinitis populations. Patients with non-allergic rhinitis demonstrated greater uncertainty regarding disease etiology, reflecting the complexity of diagnosing and understanding conditions whose triggers may be less obvious than those of allergic rhinitis.

Education is frequently overlooked as a clinical intervention, yet it may be one of the most scalable tools available for improving outcomes. Patients who understand their disease are better equipped to avoid triggers, adhere to treatment plans, recognize worsening symptoms, and engage in shared decision-making. Conversely, inadequate disease knowledge can amplify existing disparities. Improving health literacy and patient education therefore remains a critical component of advancing equity in allergic disease management. However, as highlighted by Kabir et al. [Integrating evidence-based paediatric asthma management in Australian primary care: phase I protocol for developing implementation bundles], education alone cannot lead to lasting outcomes if not integrated within a multilevel framework that includes not only patients and their families, but also their health care team and the systems in which they function.

The importance of addressing inequities at a population level is further explored in the review by Li et al. highlighting the “*Forgotten Billion*” [The forgotten billion: bridging the global allergy care chasm through scalable, equity-driven strategies]. The authors examine the burden of allergic diseases in low- and middle-income countries (LMICs), where shortages of healthcare resources, workforce limitations, and restricted access to essential therapies continue to contribute to poor outcomes.

Asthma provides one of the clearest examples of this challenge. Despite the existence of highly effective therapies, asthma continues to cause substantial morbidity and mortality worldwide, with a disproportionate burden occurring in LMICs. Access to inhaled corticosteroids, the cornerstone of persistent asthma management, remains inconsistent in many settings. The result is a persistent gap between what is scientifically possible and what patients actually receive.

Importantly, Li et al. highlight several successful strategies that have improved allergic disease outcomes across different settings, including primary-care training programs, community health worker interventions, and national policy initiatives. A key lesson from these examples is that successful interventions must be adapted to local contexts. Strategies that work in one country or healthcare system cannot simply be transplanted into another without consideration of local infrastructure, workforce capacity, and patient needs. The review also reinforces a broader point that extends beyond LMICs. Allergic diseases are too common and too widespread to be managed solely through specialist-centered models of care. While allergists and immunologists provide essential expertise, most patients interact primarily with general practitioners, pediatricians, nurses, pharmacists, and other frontline healthcare professionals. Bringing evidence-based allergy care closer to where patients live and receive routine healthcare may represent one of the most effective strategies for reducing disparities. Patient-centered models that strengthen primary care capacity are likely to generate greater population-level gains than approaches that rely exclusively on specialist access.

The opportunities and challenges associated with technology are explored in the review by Lourenço and Raposo [Digital health in allergy care: current practices, evidence, and future prospects], who examine the role of digital health in allergy care. The authors describe an expanding landscape that includes telemedicine, mobile health applications, artificial intelligence (AI), remote monitoring tools, and clinical decision support systems. Digital technologies have the potential to address several barriers that contribute to inequities in allergy care. Telemedicine can improve access to specialist expertise in rural and underserved communities. Clinical decision support tools integrated within electronic medical records can help frontline providers recognize allergic diseases earlier and implement evidence-based management strategies. Mobile applications can support medication adherence, symptom tracking, allergen exposure monitoring, and patient education. These innovations may be particularly valuable for populations that experience delays in diagnosis or reduced access to specialty care. Clinical decision support systems, for example, can assist primary care providers in recognizing conditions that may otherwise go undiagnosed or undertreated. Similarly, patient-facing technologies can improve self-management and empower patients to play a more active role in their care.

However, technology alone is not a solution. Digital tools that improve outcomes in one setting may not produce similar results elsewhere. Successful implementation requires validation, adaptation, and consideration of barriers such as internet access, language, health literacy, and digital literacy. Moreover, technology should augment, not replace, the patient-clinician relationship. A symptom-tracking application should enhance conversations about disease control rather than substitute for them. Effective allergy care remains grounded in communication, trust, and shared decision-making.

While implementation and access remain critical themes throughout this collection, therapeutic innovation continues to transform outcomes for patients with severe and rare diseases. In their review of evolving therapeutic strategies for hereditary angioedema (HAE), Sonija and colleagues describe the expanding treatment landscape, including newer agents such as donidalorsen and sebetralstat. HAE is a potentially life-threatening disease characterized by recurrent episodes of angioedema that can result in significant morbidity and, in some cases, fatal airway compromise. Historically, treatment options were limited and often burdensome. The emergence of targeted therapies has changed the management of HAE, providing patients with safer, more effective, and increasingly convenient treatment options. The development of oral on-demand therapies and long-acting prophylactic treatments represents more than a scientific achievement. These advances improve quality of life, increase patient autonomy, and enable more personalized disease management. The HAE experience illustrates the transformative potential of therapeutic innovation. At the same time, it highlights a recurring challenge in healthcare: innovation only improves outcomes when patients can access it. Ensuring equitable access to novel therapies remains essential if advances in treatment are to translate into advances in population health.

The importance of implementation is perhaps most explicitly addressed by Kabir et al. [Integrating evidence-based paediatric asthma management in Australian primary care: phase I protocol for developing implementation bundles] in their study protocol describing the development of implementation bundles for pediatric asthma management in Australian primary care. Their work focuses on a challenge familiar to clinicians across healthcare systems: guidelines exist, but adherence to those guidelines is often inconsistent. In asthma, they noted that 20% of kids hospitalized for asthma are hospitalized within a year, and that only about half of GPs document the asthma triggers, about 45% document asthma action plans, and about one-fifth note inhaler adherence- cardinal and important features in optimizing asthma care in children and preventing readmission. The authors propose a multidimensional framework, “bundles”, that addresses barriers at the patient, provider, and practice levels. Their approach recognizes that improving outcomes requires more than publishing recommendations or conducting educational sessions. Sustainable improvement depends on integrating evidence-based care into routine clinical workflows while engaging patients, families, clinicians, and healthcare organizations.

This implementation-focused approach is particularly relevant in pediatric asthma, where preventable exacerbations continue to contribute to hospitalizations and healthcare utilization. Documentation of triggers, asthma action plans, inhaler technique, and medication adherence remains inconsistent despite their recognized importance. The implementation bundles proposed by Kabir et al. seek to make guideline-concordant care easier to deliver and easier to sustain. Their work reinforces a principle that extends across allergic diseases: guidelines do not improve outcomes unless they are implemented. Implementation requires stakeholder engagement, institutional support, workflow integration, and systems to make evidence-based practice the easiest path forward.

The broader context for these challenges is provided by Mendez et al. in their review, [Advancing equity in allergy and immunology: progress, pitfalls, and the path forward]. The authors describe how social determinants of health influence allergic disease outcomes across multiple conditions and throughout the continuum of care.

Underserved populations frequently experience barriers related to healthcare access, environmental exposures, affordability of medications, and availability of specialty services. These barriers contribute not only to delays in diagnosis and treatment but also to increased disease severity and poorer outcomes. Such disparities are evident across numerous allergic and immunologic conditions, including asthma, food allergy, atopic dermatitis, and primary immunodeficiencies.

Importantly, the review highlights opportunities to address these inequities through policy, legislation, and community engagement. Environmental regulations, efforts to improve medication affordability, expansion of access to lifesaving therapies, and patient advocacy initiatives all have the potential to improve outcomes at a population level. Equally important are community-based support systems and patient networks that help individuals navigate healthcare systems and advocate for their needs.

Taken together, the six articles in this Research Topic underscore that advancing equity in allergic disease management requires action at multiple levels. Education, implementation science, digital innovation, therapeutic advances, primary care engagement, and public policy are not competing priorities; they are complementary strategies. No single intervention will eliminate disparities in allergic disease outcomes. Rather, progress will require coordinated efforts that address barriers experienced by patients, providers, healthcare systems, and communities.

The central message emerging from this collection is clear. The future of allergy and immunology will be shaped not only by scientific discoveries but also by our ability to ensure that those discoveries benefit all populations. The challenge is no longer simply generating evidence. It is implementing evidence. It is bringing care closer to patients. It is ensuring that innovation reaches underserved communities as effectively as it reaches well-resourced ones. Ultimately, advancing equity in allergic disease management requires moving beyond what is possible scientifically and focusing on what is achievable for patients in the real world.

